# Supplementary material for: Factors motivating female sex workers to initiate pre- exposure prophylaxis for HIV prevention in Zimbabwe
Source: PLoS One. 2022 Jul 7;17(7):e0264470. doi: 10.1371/journal.pone.0264470 (PMC9262226; doi:10.1371/journal.pone.0264470)
Supplement: Attachment — Submitted filename: Response to Reviewers.docx [file pone.0264470.s002.docx]

**Response to Reviewer’s comments**

Please use the space provided to explain your answers to the questions above. You may also include additional comments for the author, including concerns about dual publication, research ethics, or publication ethics. (Please upload your review as an attachment if it exceeds 20,000 characters)

Reviewer #1: Good paper with sound methods and analysis. Very minor edits to be addressed below. I'm curious to know about the engagement with the clinic. Do the FSW come on a routine basis? Not sure if this will even impact the study, just felt like an unanswered question that could potentially lead to the sampling basis being a set of women who have stronger health seeking behaviors than fellow FSW who don't engage with the clinic.  Thank you for the comment. We have added that the sex workers routinely frequent the clinic for various sexual reproductive health (SRH) including HIV prevention services, line 128.

I would also consider removing the number of children simply as there could be the possibility (although unlikely) of this set of women being identified as you noted the location of the clinic serving FSW already. There likely aren't too many clinics like this. Thank you for the comment. We agree and have removed the number of children on all quotations used in the manuscript as this could be an identifier (Line 247, 268, 266, 273, 295, 301, 305, 322, 326, 330, 348, 354, 358, 374, 378, 383, 402, 406, 415, 429, 433, 439, 454.

Would also recommend a table of participant characteristics in the beginning with ranges such as ages (ex. 18-25, 26-34, 34+), number of kids (ex. 1, 2-3, 4-5, 6+) etc. and then have these ranges with the quotes to help de-identify respondents even more. Such a table would also get the reader familiar with the characteristics of the women as well.  Thank you for your comment. We have added a demographic table at the beginning of the findings section (line 224). We also added age ranges and removed the number of children as identifiers on all quotations used in the manuscript.

I find it important to discuss more the fact that women seem to transition from study to study for PrEP. It is noted that it's an interesting finding, yet it seems to not be followed up on at all. Thank you for the comment. Line 491 speaks about post-trial access for FSWs participating in clinical trials. We have added the following “Our findings reveal that some FSWs transition from one HIV prevention trial to the next to enable access to HIV prevention. Clinical trials were also reported to deliver free quality services, with clients reporting to be treated respectfully compared to public health facilities” to bring out the point.

In addition, I'm curious as to why quantitative studies would help better understand what factors motivate FSW; I would think, if anything, quantitative would only be involved in some kind of mixed method study to have qualitative data elucidate any findings related to barriers. But, quantitative could be the way! Would just clarify why this was specifically put in a qualitative paper. Thank you for the comment. We have removed the statement as the paper identifies factors motivating FSWs to initiate PrEP.

Reviewer #2: Line 133 translated and transcribed?   Thank you for the question. The pilot data was transcribed and translated and included as part of the research data. Line 196

Line 170 – please explain what bracketing is and how it is used  . Thank you for the comment. We have added the following, bracketing is a qualitative approach used to minimize the potentially damaging effects of preconceptions that may taint the research process. This was achieved through researcher reading and re-reading the transcripts to fully engage with the data as well as through the use of direct quotations from the FSWs themselves to explain their motivations for initiating on PrEP. We have also added an additional citation (Tufford & Newman, 2012). Line 207.

Line 226-228 – there seems to be a theme around children there, perhaps you can move that quote down to the relevant section 315 - …. and find another quote for protection only  . Thank you for the comment. We have moved the quote from quote to section 315. We have identified a quote that speaks to protection only. “…*As sex workers, we meet different clients and we do not know their HIV status so, it is better for us to be on PrEP and protect ourselves”.( Tess, P15, 18-24 years) line 282*

Condom bursting comes through in quotes throughout the paper as a concern that is allayed by PrEP, does this not count as its own motivating theme? Perhaps – reliability of current HIV prevention methods  . Thank you for the comment. We agree that condom bursting comes through in quotes throughout the paper and we have now included it as a theme on it’s own. Condoms bursting-One frequently occurring concern from the FSWs was the condoms bursting due to their clients being drunk or using force during sex. This issue is exemplified by the following quotes..line 293

*We meet a lot of different clients and you find that at times condoms burst.., (Tsitsi, P1, 36-45years)*

*Some of our clients can be very violent such that the condom itself may end up breaking. In such cases, if you are on PrEP at least you know you are protected (Nyasha P5, 36-45years)*

How did you classify internal vs external motivation? There is a thin line and a sentence or so on how these were defined will help. We have included a definition of intrinsic and external motivation and a citation as follows in line 230; While intrinsic motivation refers to FSWs taking PrEP because they find it rewarding in itself, extrinsic motivation is driven from an external force with an expectation to receive some perceived rewards (Tranquillo & Stecker, 2016).

I would assume that the money aspect is internal for survival or self actualisation in starting a business so delineation would help.   We have defined how external and internal motivators are used in the paper , line 230 and feel that the money aspect still fits as an external motivating factor. FSWs could have unprotected sex to get more money from their clients.

The theme of FSWs being that for a season to raise money to start a business is interesting and am curious to see if there were more FSWs that wanted to stay negative for when they leave the profession.   Some of the younger FSWs raised the issue of wanting to start a business as they saw sex work as a temporary measure to meet some of their financial needs. We have added the following. “Some of the younger FSWs viewed sex work as a temporary measure to meet their financial needs” line 343.

Line 291 – who is the “she” referred to in the quote? It helps to know the relationship between the client and “she”. Thank you for the comment. The “She” refers to the participant’s mother and we have changed this to make it clear for the reader, line 373

Peer influence or parental like in the quote above  Line 297 – does helped there mean offered PrEP, this may be local context lingo of helped but it may not be easily understood internationally  . Thank you for the comment. We have changed the language to reflect that “helped” means – after the participant had been seen by the nurse line 385.

Line 307-309 – seems to reveal the underlying reason for staying on PrEP, yes the initial introduction was in trials but her motivation was GBV or her SDC dynamics and how her man is not acting in her best interests. Can you pull that out as its own theme?  Thank you for the comment. We agree with you and now have GBV as a separate theme. We have included the following under the new them GBV- **Some participants reported experiences of GBV with some living with partners who were living with HIV, hence the need to protect themselves through PrEP line 411.**

Line 365 – I believe the word should be imminent not eminent  . This is well noted and have made the change line 488.

The paper is interesting because it highlights more than the obvious reasons for FSW PrEP demand. I would like the researchers to draw parallels between their findings and papers from women in SDCs for example and how both groups can be targeted together or how the service delivery approaches differ. This is noted and we have included the paragraph below to highlight some of the similarities and differences between FSWs and SDCs as well as how the two population groups may be targeted using similar strategies. We have included the following;

Whilst there may be some similarities in motivational factors for PrEP initiation among FSWs and other population groups (9–13,15), including sero-discordant couples (SDCs) whose motivation for PrEP is to remain HIV negative whilst preserving their relationships (33,34), it must be noted that FSWs are a unique population group that require tailor-made strategies and interventions to ensure approaches are amplified and are delivered on a larger scale for population-level impact (35). Line 513

May the researchers please include some suggestions/recommendations for how their findings can be used to better target sex workers for HIV prevention? Ads that highlight benefits to sex workers? How will these ads differ from women in SDCs that take PrEP for the same reasons and have a similar high risk? This is well noted. We have included a paragraph to draw parallels between FSWs and SDCs. We have included the following paragraph: - Given the similarities in HIV risk between FSWs and SDCs, some of the PrEP demand creation materials and messaging may equally apply to both groups as the overriding factor is to remain HIV negative. Line 522.
