# Supplementary material for: Factors motivating female sex workers to initiate pre- exposure prophylaxis for HIV prevention in Zimbabwe
Source: PLoS One. 2022 Jul 7;17(7):e0264470. doi: 10.1371/journal.pone.0264470 (PMC9262226; doi:10.1371/journal.pone.0264470)
Supplement: Attachment — Submitted filename: Comments.docx [file pone.0264470.s003.docx]

**Dear Editor,**

Thank you for providing me the opportunity to review this manuscript. There are many concerns which are as fallow:

**Title:**

1) The title is not reflecting the aim of the study.

2) It is strongly suggested to consider the study design in the title.

**Abstract:**

1) The aim is “to understand factors that motivate FSWs to initiate PrEP, in Harare, Zimbabwe” while the tile seem is about if participants are motivated or not.

2) This sentence needs English edit “Data was analyzed using Colaizi’s seven steps to analyze DPA data.”.

3) In the results section, it has mentioned that 7 themes were extracted, according to my knowledge the themes should be broader and more limited in number. It seems these are subthemes.

**Key word:**

1) there was no keyword.

**Introduction:**

1) Lines 47- 48, the reference 3 is not cited in the text.

2) There is no explanation about the context.

**Method:**

1) While estimating sample size for qualitative research is mentioned in some literature, however it is not enough and it will be finalized by data saturation. Please explain about it.

2) Line 120, why it has referenced to other study?

3) Only one month has dedicated to sampling? How it is possible? In the qualitative research the sampling and data analysis are conducted simultaneously to better manage the interview process and the study. It seems that the authors interviewed all participants and then analyzed the data.

4) Who were responsible for interview and how she/he were trained for interviewing?

5) Line 139, why it has referenced to other studies?

6) Please report the interview guide.

7) Please move “Member checking involves the taking of findings back to study participants to ensure data has been interpreted correctly taking account of the context” to the rigor section.

8) Please explain about external validity and maximum variation.

9) Please explain about the time of data analyzing, and the software which has used for data managing.

10) Please report an example for the data analysis process.

**Results:**

1) I believe the authors has wrongly used the word “theme”.

2) “Fear of getting infected with HIV and condoms bursting” is not reported in the abstract.

3) I see no different between the “Self-protection from HIV infection and fear for one’s health” and “Fear of getting infected with HIV” subthemes.

4) according to the quotations, I believe “Increased financial freedom as a motivator to initiate PrEP” could be replace with “Increased the chance for offering unprotected sex”.

5) The “Gender based Violence (GBV)” is not well explained and even though the quotation is no related.

“When both studies came to an end, my boyfriend, who is HIV positive, was now staying with me (at my house) permanently. So, when the study ended, l just saw it fit that l should go on PrEP because of his cruelty to me. Sometimes he would boil my family planning pills and hide my female condoms. (Fortu, P2, 36-45 years)”!!

6) I believe the results are not rich.

**Discussion**

1) Line 367- 368, why it has referenced to other studies?

2) The first paragraph of the discussion is a repetition of introduction.

3) The discussion should be more concise and need more interpretation and comparing with evidence.

4) When we seek the participants experience it is not related when he/she has experienced it. On the other had it is a major concern of the present study which did not considered different experience and did not consider maximum variation in sampling.
