# Supplementary material for: Factors motivating female sex workers to initiate pre- exposure prophylaxis for HIV prevention in Zimbabwe
Source: PLoS One. 2022 Jul 7;17(7):e0264470. doi: 10.1371/journal.pone.0264470 (PMC9262226; doi:10.1371/journal.pone.0264470)
Supplement: Attachment — Submitted filename: Response to Reviewers.docx [file pone.0264470.s005.docx]

**Dear Editor,**

**Please find in the table below our individual responses to the comments raised by reviewer 1 and 2.**

| **Reviewer 1:** Reviewer #1: This is a simple, straightforward , qualitative ,well written research paper on intrinsic and extrinsic factors that motivate women in sex work to start PrEP. It uses the seven steps of Colaizzi's method for data analysis which is a well-known method used in qualitative research. I really don't have anything new to add to this paper other than saying that since this was a small group of women (20) the findings and experiences could have been corroborated further with a Focus Group Discussion, but I guess this was not possible as the topic of sex work is a sensitive one and requires confidentiality. Some corrections need to be done in this paper as follows : | | |
| --- | --- | --- |
| **Number** | **Comment** | **Response** |
| **1** | . Line 33 - the first point for intrinsic factor is expressed clearly through a (i). The second point -fear for one's health should also be preceded by (ii) | Thank you and this has been done. We have added (ii) |
| **2** | Line 106- The word illicit should be replaced by elicit. | We agree, the word illicit has been replaced with elicit. |
| **3** | Line 231- the first word in this line is not clear. | This has been explained “Haa, meaning – pausing to think |
| **4** | Lines 316-320 and lines 330 -333 are identical to each other though used in different context. | We have replaced the quote in line 330 with another quote. |
| **5** | Table -1 The item column should be clearly elucidated. n should be numbers, and percentage followed by % symbol. | Thank you, the table has been relabeled to make it clearer. We now have the first column labeled as Item, second column sub-category, the third column – N=20, fourth column – percentage (%). |
|  |  |  |
| **Reviewer 2:** Thank you for providing me the opportunity to review this manuscript. There are many concerns which are as fallow: | | |
| **Title:** |  |  |
| **1** | The title is not reflecting the aim of the study. | We have changed the title to “Factors motivating female sex workers to initiate Pre-exposure Prophylaxis for HIV Prevention in Zimbabwe”. |
| **2** | It is strongly suggested to consider the study design in the title. | We have changed the title to “Factors motivating female sex workers to initiate Pre-exposure Prophylaxis for HIV Prevention in Zimbabwe”. |
| **Abstract:** |  |  |
| **1** | The aim is “to understand factors that motivate FSWs to initiate PrEP, in Harare, Zimbabwe” while the tile seem is about if participants are motivated or not. | This has been addressed in the title section above. The title has been revised to “Factors motivating female sex workers to initiate Pre-exposure Prophylaxis for HIV Prevention in Zimbabwe”. |
| **2** | This sentence needs English edit “Data was analyzed using Colaizzi’s seven steps to analyze DPA data.”. | We agree and this has been revised to “Data was analyzed using Colaizzi’s seven steps for analyzing data. |
| **3** | In the results section, it has mentioned that 7 themes were extracted, according to my knowledge the themes should be broader and more limited in number. It seems these are subthemes. | We agree with this comment, we have clarified it and it now says, “Two broad themes were identified namely extrinsic and extrinsic motivators. The two broad themes each has several sub-themes with three sub-themes under intrinsic motivation and six sub-themes under extrinsic motivation. |
| **Key word:** |  |  |
| **1** | there was no keyword. | We have added the following key words:  Pre-exposure Prophylaxis, PrEP, PrEP adherence, Female sex workers, Descriptive Phenomenological Analysis (DPA), PrEP initiation, PrEP motivation. |
| **Introduction:** |  |  |
| **1** | 1) Lines 47- 48, the reference 3 is not cited in the text. | The reference has been cited in the text. It is a systematic review on the burden of HIV among female sex workers. This has been added to line 47-48 where we described the burden of HIV among female sex workers. |
| **2** | There is no explanation about the context. | The context is within the female sex worker’s burden of HIV and has been explained in (1) above. We have added the following statement to explain the context – “Female sex workers in Zimbabwe are at heightened risk of HIV with an estimated HIV incidence of 10% against a national HIV incidence of 0.47% (1,2) |
| **Method:** |  |  |
| **1** | While estimating sample size for qualitative research is mentioned in some literature, however it is not enough and it will be finalized by data saturation. Please explain about it. | In qualitative research sample sizes estimations are used to give guidance on the number of participants to be interviewed but, the actual number is determined by when the researcher reaches data saturation. We have added the following citations (3,4) |
| **2** | Line 120, why it has referenced to other study? | We have added “in line with the Medical Research Council of Zimbabwe (MRCZ) guidance” This is the institution review board (IRB) guidance that is referenced. |
| **3** | Only one month has dedicated to sampling? How it is possible? In the qualitative research the sampling and data analysis are conducted simultaneously to better manage the interview process and the study. It seems that the authors interviewed all participants and then analyzed the data. | Thank you for the comment and we agree there was an error in the text. There was an error where it said ‘Data was collected in March 2020” We have corrected that to reflect that “Data was collected from March 2020”. This process started in March and was completed in August 2020. |
| **4** | Who were responsible for interview and how she/he were trained for interviewing? | We have added this –“The first author conducted all the 20 interviews from March 2020 – August 2020. The first author received numerous trainings on how to conduct qualitative research including receiving training on how to conduct descriptive phenomenological analysis. The first author received guidance and support from the second author who was the supervisor and a Professor with at least two decades of experience in conducting qualitative research”. |
| **5** | Line 139, why it has referenced to other studies? | This was referenced to show the work that was done before. We have included the following statement “as done in previous studies”. |
| **6** | Please report the interview guide. | We agree and have now added the following text in the interview guide section: “The interview guide was developed by the first author in consultation with the second author who has more than two decades of developing qualitative guides. The interview guide was developed to be responsive to the research objective which was aimed at exploring factors motivating female sex workers to initiate PrEP”. |
| **7** | Please move “Member checking involves the taking of findings back to study participants to ensure data has been interpreted correctly taking account of the context” to the rigor section. | We agree and member checking has been moved to the rigor section. |
| **8** | Please explain about external validity and maximum variation. | Thank you for the comment. In our qualitative paper, we have addressed this through trustworthiness “To ensure trustworthiness, the first author took the preliminary findings back to the FSWs who had participated in the interviews to check if the data had been interpreted in the correct way and as intended by the FSWs. |
| **9** | Please explain about the time of data analyzing, and the software which has used for data managing. | We have included the following to address the time of data analysis and software used to manage the data. ‘Each interview was transcribed within 24 hours of conducting the interview. Nvivo was used to manage the data and extract and classify FSW phrases, thereby making it easier to identify emerging themes across the FSW motivations as each additional FSW was recruited. |
| **10** | Please report an example for the data analysis process. | Data was analysed in line with Colaizzi’s seven steps of DPA approach and a spreadsheet with some of the emerging themes has been included for reference. |
| **Results:** |  |  |
| **1** | I believe the authors has wrongly used the word “theme”. | Thank you for this comment. We agree that we have wrongly used the word “theme”. We have included the following wording “The findings revealed two major themes namely intrinsic and extrinsic motivations. These two major themes each had several sub-themes with intrinsic motivational factors having three sub-themes and extrinsic motivational factors having six sub-themes. |
| **2** | “Fear of getting infected with HIV and condoms bursting” is not reported in the abstract. | These have now been included in the abstract and this text have been added, “Two themes were identified as intrinsic and extrinsic motivations. Intrinsic motivations included (i) self-protection from HIV infection (ii) condoms bursting. Extrinsic motivators included (i) occupational risk associated with sex work (ii) increased chance of offering unprotected sex as a motivator to initiate PrEP (iii) positive encouragement from others (iv) need to take care of the children (v) prior participation in HIV prevention research studies (vi) GBV |
| **3** | I see no different between the “Self-protection from HIV infection and fear for one’s health” and “Fear of getting infected with HIV” subthemes. | These themes have now been combined under one sub-theme. |
| **4** | according to the quotations, I believe “Increased financial freedom as a motivator to initiate PrEP” could be replace with “Increased the chance for offering unprotected sex”. | We agree and the sub-theme – increased financial freedom as a motivator to initiate PrEP” has been renamed increased chance of offering unprotected sex as a motivator for initiating PrEP” |
| **5** | The “Gender based Violence (GBV)” is not well explained and even though the quotation is no related.  “When both studies came to an end, my boyfriend, who is HIV positive, was now staying with me (at my house) permanently. So, when the study ended, l just saw it fit that l should go on PrEP because of his cruelty to me. Sometimes he would boil my family planning pills and hide my female condoms. (Fortu, P2, 36-45 years)”!!. | We have now explained GBV as follows – Gender Based Violence is defined as any violence against women that results in or is likely to result in physical, sexual or psychological harm or suffering to women (5). Gender Based Violence was reportedly experienced by some FSWs as a result of their being on PrEP. One FSW reported that “my boyfriend would beat me up saying he did not want to see me taking those pills as they would encourage her to sleep with other men”(Tsitsi P1, 36-45 years) |
| **6** | I believe the results are not rich. | Thank you for the comment. We believe by addressing each one of the comments raised and further collapsing some of the seemingly related sub-themes, we have strengthened and improved the richness of the findings. |
| **Discussion** |  |  |
| **1** | Line 367- 368, why it has referenced to other studies? | We agree this statement does not need any reference to prior studies and we have removed the references. |
| **2** | The first paragraph of the discussion is a repetition of introduction. | We agree and we have replaced the first paragraph with the following text.  “The objective of the study was to identify factors that motivate FSWs to initiate PrEP using DPA as the methodology. The findings reveled two main themes namely intrinsic and extrinsic motivational factors. Intrinsic factors included self-protection from HIV infection condoms bursting whilst extrinsic motivators included occupational risk associated with sex work, increased chances of offering unprotected sex, positive encouragement from others, prior participation in PrEP studies, GBV and the need to take care of children”. |
| **3** | The discussion should be more concise and need more interpretation and comparing with evidence. | We have included the following text as part of the discussion section,  Our findings suggest that factors motivating FSWs to initiate on PrEP include both intrinsic and extrinsic motivational factors. Despite WHO guidance recommending that all population groups with an HIV incidence of 3% or higher be prioritized for PrEP, PrEP initiation requires further motivation beyond the elevated HIV risk perception (6).  Violence, including gender-based violence (GBV) emerged as a motivator for initiating on PrEP. Violence has been associated with HIV risk across different population groups. This finding is consistent with a study among PBFW which reported forced sex and GBV as motivational factors for PrEP initiation (7). In a study among men who have sex with men (MSM), who reported experiencing physical, emotional and sexual violence frequently, violence was a major motivator for MSM to initiate on PrEP to ensure they are protected from HIV (8).  Our findings also suggest that FSWs experience motivators that may be unique to them as part of their work as sex workers which other population groups may not experience. Some of the PreP motivational factors which may be unique to FSWs included positive encouragement from other FSWs and having increased chances of offering unprotected sex as they will feel they are protected from HIV. These motivational factors suggest the need to develop psychosocial interventions to better support FSWs to initiate PrEP. |
| **4** | When we seek the participants experience it is not related when he/she has experienced it. On the other had it is a major concern of the present study which did not considered different experience and did not consider maximum variation in sampling. | Thank you for the comment. Female sex workers were individually recruited as they came to the clinic and with different experiences. We used the eligibility criteria which was set as follows:  Identify as a FSW, had been initiated on PrEP and had visited the specialized clinic at the time of enrollment.  In qualitative research, we normally do not use “maximum variation” as applied in quantitative research sampling. In this qualitative research sampling, the eligibility criteria was applied and this ensured that FSWs in their diversity could be eligible as long as they had been initiated on PrEP. The characteristics of the FSWs are evidenced by the demographic characteristics of the FSWs that were included in the study from line 180-190. |
|  |  |  |
|  |  |  |

References

1.	Cowan FM, Davey C, Fearon E, Mushati P, Dirawo J, Chabata S, et al. Targeted combination prevention to support female sex workers in Zimbabwe accessing and adhering to antiretrovirals for treatment and prevention of HIV (SAPPH-IRe): a cluster-randomised trial. Lancet HIV [Internet]. 2018 Aug [cited 2020 Mar 10];5(8):e417–26. Available from: https://linkinghub.elsevier.com/retrieve/pii/S2352301818301115

2.	ZIMBABWE POPULATION-BASED HIV IMPACT ASSESSMENT ZIMPHIA 2020 KEY FINDINGS [Internet]. 2020 [cited 2021 Mar 10]. Available from: https://phia.icap.columbia.edu/wp-content/uploads/2020/11/ZIMPHIA-2020-Summary-Sheet_Web.pdf

3.	Sebele-Mpofu FY. Saturation controversy in qualitative research: Complexities and underlying assumptions. A literature review. Serpa S, editor. Cogent Soc Sci [Internet]. 2020 Jan 1 [cited 2022 Apr 13];6(1):1838706. Available from: https://www.tandfonline.com/doi/full/10.1080/23311886.2020.1838706

4.	Mason M. Sample Size and Saturation in PhD Studies Using Qualitative Interviews. Forum Qual Sozialforsch / Forum Qual Soc Res [Internet]. 2010 Aug 24 [cited 2022 Apr 13];11(3). Available from: https://www.qualitative-research.net/index.php/fqs/article/view/1428

5.	Bott S, Morrison A, Ellsberg M. Preventing and responding to gender-based violence in middle and low-income countries: a global review and analysis. World Bank Res Work Pap 3618 [Internet]. 2005 [cited 2022 Apr 13];(June):61. Available from: https://books.google.co.za/books?hl=en&lr=&id=bHuoNOV1MGUC&oi=fnd&pg=PA3&dq=gender+based+violence+definitions&ots=tN__6OLEHL&sig=bar6NRoct_q_Qx538v9p0N2DOMA&redir_esc=y#v=onepage&q=gender based violence definitions&f=false

6.	World Health Organization (WHO). THE USE OF ANTIRETROVIRAL DRUGS FOR TREATING AND PREVENTING HIV INFECTION 2016 [Internet]. 2016 [cited 2021 Aug 19]. Available from: https://apps.who.int/iris/bitstream/handle/10665/208825/9789241549684_eng.pdf;jsessionid=361EE1EB5A7FC64282355F2BF0C3B27A?sequence=1

7.	Kinuthia J, Pintye J, Abuna F, Mugwanya KK, Lagat H, Onyango D, et al. Pre-exposure prophylaxis uptake and early continuation among pregnant and post-partum women within maternal and child health clinics in Kenya: results from an implementation programme. Lancet HIV [Internet]. 2020 Jan 1 [cited 2021 Aug 9];7(1):e38–48. Available from: https://www.sciencedirect.com/science/article/abs/pii/S2352301819303352

8.	Pintye J, Rogers Z, Kinuthia J, Mugwanya KK, Abuna F, Lagat H, et al. Two-Way Short Message Service (SMS) Communication May Increase Pre-Exposure Prophylaxis Continuation and Adherence Among Pregnant and Postpartum Women in Kenya. Glob Heal Sci Pract [Internet]. 2020 Mar 30 [cited 2021 Apr 5];8(1):55–67. Available from: http://www.ncbi.nlm.nih.gov/pubmed/32139420

Yours Sincerely

Definate Nhamo
