# Supplementary material for: Factors motivating female sex workers to initiate pre- exposure prophylaxis for HIV prevention in Zimbabwe
Source: PLoS One. 2022 Jul 7;17(7):e0264470. doi: 10.1371/journal.pone.0264470 (PMC9262226; doi:10.1371/journal.pone.0264470)
Supplement: Attachment — Submitted filename: Response to Reviewers.docx [file pone.0264470.s006.docx]

**Dear Editor,**

**Please find in the table below our individual responses to the comments raised by reviewer 1.**

Reviewer #1: the paper is well written and explained in great detail.
some corrections :

1. Line 32 under abstract - the line should read intrinsic and extrinsic factors .
2. Line 96- when expanding DPA for the first time , the first letter of each word should be in capitals
3. Table one - please add age in years under item
4. Line 406- Several abbreviations are mentioned -kindly expand

| Reviewer #1: the paper is well written and explained in great detail. some corrections : | | |
| --- | --- | --- |
| **Number** | **Comment** | **Response** |
| **1** | Line 32 under abstract - the line should read intrinsic and extrinsic factors. | Thank you for the comment. We have added ”intrinsic and extrinsic factors”. |
| **2** | Line 96- when expanding DPA for the first time , the first letter of each word should be in capitals. | Thank you for the comment, we have changed this to “Descriptive Phenomenological Analysis”. |
| **3** | Table one - please add age in years under item. | Thank you, we have added “Age in years” under item. |
| **4** | Line 406- Several abbreviations are mentioned -kindly expand. | Thank you, we have expanded the abbreviations as follows;  Pre-Exposure Prophylaxis (PrEP)  Female Sex Workers (FSWs)  The World Health Organization (WHO) |
|  |  |  |

Yours Sincerely

Definate Nhamo
